# Identification and gene expression profiling of human gonadotrophic pituitary adenoma stem cells

**DOI:** 10.1186/s40478-023-01517-w

**Published:** 2023-02-07

**Authors:** Linhao Yuan, Peiliang Li, Jiang Li, Jiayi Peng, Jianlong Zhouwen, Shunchang Ma, Guijun Jia, Wang Jia, Peng Kang

**Affiliations:** 1 Department of Neurosurgery, Beijing Tiantan Hospital, Capital Medical University, Beijing, China; 2 China National Clinical Research Center for Neurological Diseases, Fengtai, Beijing, China; 3 Beijing Neurosurgical Institute, Beijing, China; 4 Department of Neurosurgery, Beijing Ditan Hospital, Capital Medical University, Chaoyang District, Beijing, China

**Keywords:** Gonadotrophic pituitary adenoma, Tumor stem cells, RNA-sequencing

## Abstract

**Background:**

Gonadotrophic pituitary adenoma is a major subtype of pituitary adenoma in the sellar region, but it is rarely involved in the hypersecretion of hormones into blood; thus, it is commonly regarded as “non-functioning.” Its tumorigenic mechanisms remain unknown. The aim of this study was to identify human gonadotrophic pituitary adenoma stem cells (hPASCs) and explore the underlying gene expression profiles. In addition, the potential candidate genes involved in the invasive properties of pituitary adenoma were examined.

**Methods:**

The hPASCs from 14 human gonadotrophic pituitary adenoma clinical samples were cultured and verified via immunohistochemistry. Genetic profiling of hPASCs and the matched tumor cells was performed through RNA-sequencing and subjected to enrichment analysis. By aligning the results with public databases, the candidate genes were screened and examined in invasive and non-invasive gonadotrophic pituitary adenomas using Real-time polymerase chain reaction.

**Results:**

The hPASCs were successfully isolated and cultured from gonadotrophic pituitary adenoma in vitro, which were identified as positive for generic stem cell markers (Sox2, Oct4, Nestin and CD133) via immunohistochemical staining. The hPASCs could differentiate into the tumor cells expressing follicle-stimulating hormone in the presence of fetal bovine serum in the culture medium. Through RNA-sequencing, 1352 differentially expressed genes were screened and identified significantly enriched in various gene ontologies and important pathways. The expression levels of ANXA2, PMAIP1, SPRY2, C2CD4A, APOD, FGF14 and FKBP10 were significantly upregulated while FNDC5 and MAP3K4 were downregulated in the invasive gonadotrophic pituitary adenomas compared to the non-invasive ones.

**Conclusion:**

Genetic profiling of hPASCs may explain the tumorigenesis and invasiveness of gonadotrophic pituitary adenoma. ANXA2 may serve as a potential therapeutic target for the treatment of gonadotrophic pituitary adenoma.

**Supplementary Information:**

The online version contains supplementary material available at 10.1186/s40478-023-01517-w.

## Introduction

Pituitary adenoma accounts for approximately 15% of primary intracranial neoplasms and is generally considered to be benign [1]. Gonadotrophic lineage adenoma, the second most common subtype of pituitary adenoma following lactotroph lineage [2], can express follicle-stimulating hormone (FSH) or leucine hormone (LH) that may not be hypersecreted into the blood. The most common clinical symptom of gonadotrophic pituitary adenoma is compression to the adjacent structures such as the optic chiasma or invasion of the cavernous sinus [3]. Therefore, this subtype of pituitary adenoma is often considered “non-functioning.” The mechanisms of gonadotrophic pituitary adenoma tumorigenesis are still undefined, which poses challenges to the development of effective adjuvant therapy following surgical resection.

Tumor stem cells (TSC) are identified as a specific subpopulation of cells possessing stem-cell properties in certain types of bulky tumors [4, 5]. TSC have been shown to manifest a certain degree of resistance to radio- or chemotherapy, which is one possible reason for tumor relapse and metastasis [6]. Presumably, they arise from the normal stem/progenitor cells in the postnatal organs [7]. In humans, single-cell transcriptomic analysis discovered that the developing pituitary gland has a major stem cell population [8]. Accumulating evidence has pointed to a specific subpopulation of pituitary stem or progenitor cells that may give rise to pituitary tumors [9]. Previously, Xu isolated human pituitary adenoma stem cells (hPASCs) and verified their cellular characteristics, including sphere-forming, self-renewal, and multipotency [10]. Compared to differentiated daughter cells, these adenoma stem-like cells could express increased levels of stemness-associated genes, anti-apoptotic proteins, and pituitary progenitor cell markers and form tumors in the mouse model [10]. Later, Wurth characterized more cellular properties of hPASCs in somatotropinoma and non-functioning pituitary adenoma [11].

To date, research on pituitary adenoma stem cells has been conducted mainly in somatotropinoma and non-functioning adenoma [12], while very few studies have been performed on other subtypes. Here, we isolated and cultured hPASCs from human gonadotrophic pituitary adenoma samples resected during neurosurgery. The aim was to elucidate the genetic profiling of hPASCs via RNA-sequencing and to further test potential candidate genes in invasive gonadotrophic pituitary adenoma. Using this in vitro hPASC model, we hope to achieve a better understanding of the tumorigenesis and invasive behavior of gonadotrophic pituitary adenoma.

## Materials and methods

### Patients and samples

Fourteen human pituitary adenoma samples obtained during neurosurgery in our department were used for hPASC culture and sequencing. The detailed clinical information of each patient is shown in Additional file 1: Table S1.

Clinically, invasive pituitary adenoma can be defined using the Knosp grade on magnetic resonance imaging [13]. If the edge of the tumor is extended beyond the lateral tangent of the intra- and supra-cavernous internal carotid artery, it is defined invasive and the grade will be 3 or 4. Wherease, non-invasive tumors were located within the tangent of the internal carotid artery and categorized as grades 0, 1 and 2 [13]. Seven non-invasive and nine invasive gonadotrophic pituitary adenoma samples were used for the verification of candidate gene expression. The clinical information is summarized in Additional file 1: Table S2. All the samples were pathologically diagnosed as gonadotrophic pituitary adenoma by two pathologists. This study was approved by the Ethics Committee of Beijing Tiantan Hospital, and written informed consent was available for all patients.

### Culture and differentiation of hPASCs

During surgery, the pituitary adenoma specimen was resected and immediately divided into two portions. One portion was “snap-frozen” and stored in liquid nitrogen for pathological immunostaining and sequencing analysis, and the other portion was transferred to the laboratory for hPASC culture. The tumor specimen was thoroughly washed with 1X phosphate buffer saline (PBS) and minced with a sharp scalpel into small pieces under sterile conditions. The cells were collected in 10 ml Dulbecco’s modified Eagle’s Medium /F-12 (DMEM/F-12) and centrifuged at 1000 rpm for 2 min. The cell pellet was dissolved in 2 ml 1X Accutase enzyme (Stemcell Technology, USA) and incubated at 37 °C for 5 min. The dissociated cells were centrifuged at 1,000 rpm for 3 min, and the cell pellet was incubated with red blood cell lysis buffer (154 mM NH_4_Cl, 10 mM KHCO_3_, 0.1 mM EDTA, pH 7.4) for 5 min to eradicate contamination from red blood cells. After washing, the cells were dissolved and cultured in the stem cell medium composed of DMEM/F-12 supplemented with 2 mM L-glutamine, 1% penicillin–streptomycin, 1X B27 (50X, Life Technologies, USA), 20 ng/ml basic fibroblast growth factor (bFGF, Peprotech) and 20 ng/ml epidermal growth factor (EGF, Peprotech) at 37 °C with 5% CO_2_ in an incubator. The stem cell medium was refreshed every 5 days.

For differentiation, the cultured hPASCs were seeded in a 24-well plate, and the culture medium was replaced with the differentiation medium composed of DMEM/F-12 supplemented with 15% fetal bovine serum (FBS), 2 mM L-glutamine, and 1% penicillin–streptomycin. The differentiation medium was refreshed every 5 days.

### Immunohistochemistry

The hPASCs were cultured in a poly-D-lysine-coated 6-well plate for attachment, then washed with 1X PBS and fixed with 4% paraformaldehyde for 30 min at room temperature. The fixed cells were incubated in 0.3% Triton™ X-100 (Sigma-Aldrich, USA) for 15 min and washed with 1X PBS three times. The cells were blocked with 5% bovine serum albumin (BSA) for 1 h and incubated with primary antibody at 4 °C overnight. The cells were then washed with 1X PBS and incubated with secondary antibody conjugated to Alexa-Fluor 488 or Alexa-Fluor 647 (abcam, USA) at 1:1000 dilution for 1 h at room temperature. The images were captured using a Zeiss microscopic imaging system with fluorescence emission system at different magnifications. For tissue staining, the samples were sectioned at a thickness of 10 μm per slice and subjected to the above procedures.

All antibodies were purchased from Abcam unless otherwise stated. The antibodies used in the experiments were as follows: rabbit anti-Sox2 (1:800), rabbit anti-Oct4 (1:500), rabbit anti-Nestin (1:200), rabbit anti-CD133 (1:500), rabbit anti-ANXA2(1:1000), and mouse anti-FSHβ (1:800, Santa Cruz Biotechnology).

### Cell count

The cells in each culture well were washed carefully with 1X PBS before harvesting. Then, the cells were collected in a 1.5-ml tube and centrifuged at 1000 rpm for 20 s. The cell pellet was resuspended in 1-ml DMEM. For counting, 10-μl of cell suspension was added to a hemacytometer, and the number of cells in the central gridded area was manually counted under the microscope. For each sample, the total number was calculated with four separate wells, each performed in duplicate.

### Enzyme-linked immunosorbent assay (ELISA)

ELISA was performed to quantify the level of FSH in the culture medium according to manufacturer’s protocol (Elabscience, USA). Briefly, 100 μl standard or culture medium sample was added to each well and incubated for 90 min at 37 °C. The liquid was then replaced with 100 μl biotinylated detection Ab for 1 h at 37 °C. After washing, 100 μl HRP conjugate was added to the well and incubated for 30 min at 37 °C. Each well was washed thoroughly before 90 μl substrate reagent was added and incubated for 15 min at 37 °C. The reaction was stopped by adding 50 μl stop solution, and the mixture was evaluated at 450 nm using a spectrophotometer (Bio-Tek, USA).

### RNA extraction

Total RNA from the hPASCs or tumor specimen was extracted by TRIzol reagent (Invitrogen, USA) according to manufacturer’s instructions. RNA integrity was assessed using the RNA Nano 6000 Assay Kit of the Bioanalyzer 2100 system (Agilent Technologies, USA). Total RNA was stored at − 80 °C before use.

### Preparation of cDNA library and RNA-sequencing

RNA sequencing was performed by the commercial provider Novogene Co. Ltd. Briefly, first-strand cDNA was synthesized using a random hexamer primer and M-MuLV Reverse Transcriptase (RNase H). Second strand cDNA synthesis was performed using DNA Polymerase I and RNase H. After the preferential selection of 250–300-bp cDNA fragments, polymerase chain reaction (PCR) was performed using Phusion High-Fidelity DNA polymerase, universal PCR primers, and Index (X) Primer. PCR products were purified (AMPure XP system), and the quality of cDNA library was assessed on the Agilent Bioanalyzer 2100 system.

Clustering of the index-coded samples was performed on a cBot Cluster Generation System using TruSeq PE Cluster Kit v3-cBot-HS (Illumina) according to the manufacturer’s instructions. After cluster generation, the library preparations were sequenced on an Illumina Novaseq platform.

### Abundance of differentially expressed genes (DEG)

After read mapping, the counts for differentially expressed genes from each sequenced library were adjusted and analyzed using the “edgeR” package from R software (3.22.5). The *P*-values were adjusted by the Benjamini & Hochberg method. An adjusted *P*-value of 0.05 and fold change of Log_2_FC were set as the threshold for significant differential expression.

### Enrichment analysis of differentially expressed genes

Gene Ontology (GO) enrichment analysis of differentially expressed genes was implemented using the cluster Profiler R package. GO terms with corrected *p* < 0.05 were considered significantly enriched by differentially expressed genes.

The Cluster Profiler R package was used to test the statistical enrichment of differential expression genes in the pathways.

### Protein–protein interaction (PPI) network

GSE26966 dataset from GEO (Gene Expression Omnibus) contains the expression profiles of 14 gonadotrophic pituitary adenomas in comparison to 3 normal pituitary tissues in the published research [14]. The differentially expressed genes of hPASCs were selected according to the following criteria: Log_2_FC > 1.5 or <  − 1.5, and adjusted *p* < 0.05. The genes presented in both datasets were illustrated using a Venn diagram, which were further subjected to PPI analysis using the STRING database (www.string-db.org). The network was outlined by Cytoscape software (3.9.1).

### Real-time PCR

First-strand cDNA was synthesized by combining total RNA (2 μg) with a reverse transcription mixture (RevertAid™ First Strand cDNA Synthesis Kit, Thermo-fisher, USA) in a total reaction volume of 20 μl, according to the manufacturer’s instructions. The mixture was incubated at 42 °C for 60 min and 70 °C for 10 min and then returned to ice. One microliter of the mixture was extracted for Real-time PCR quantification together with 25-μl SYBR Green PCR Master Mix (TaKaRa, Japan) in a TaKaRa 7500 Real-time system (TaKaRa, Japan). β-Actin served as the internal control. The relative expression level was quantified using the 2^−∆∆ct^ method. The primers used for qPCR are mentioned in Additional file 1: Table S3.

### Statistical analysis

Statistical analyses were performed using GraphPad Prism 6.0 software (GraphPad Software, Inc., USA). Data are shown as the mean ± standard error of the mean (SEM) or the mean ± standard deviation (SD). A *t*-test was used to compare the statistical differences. A *P*-value < 0.05 was considered to indicate a significant difference.

## Results

### Culture and verification of hPASCs

**Fig. 1:**
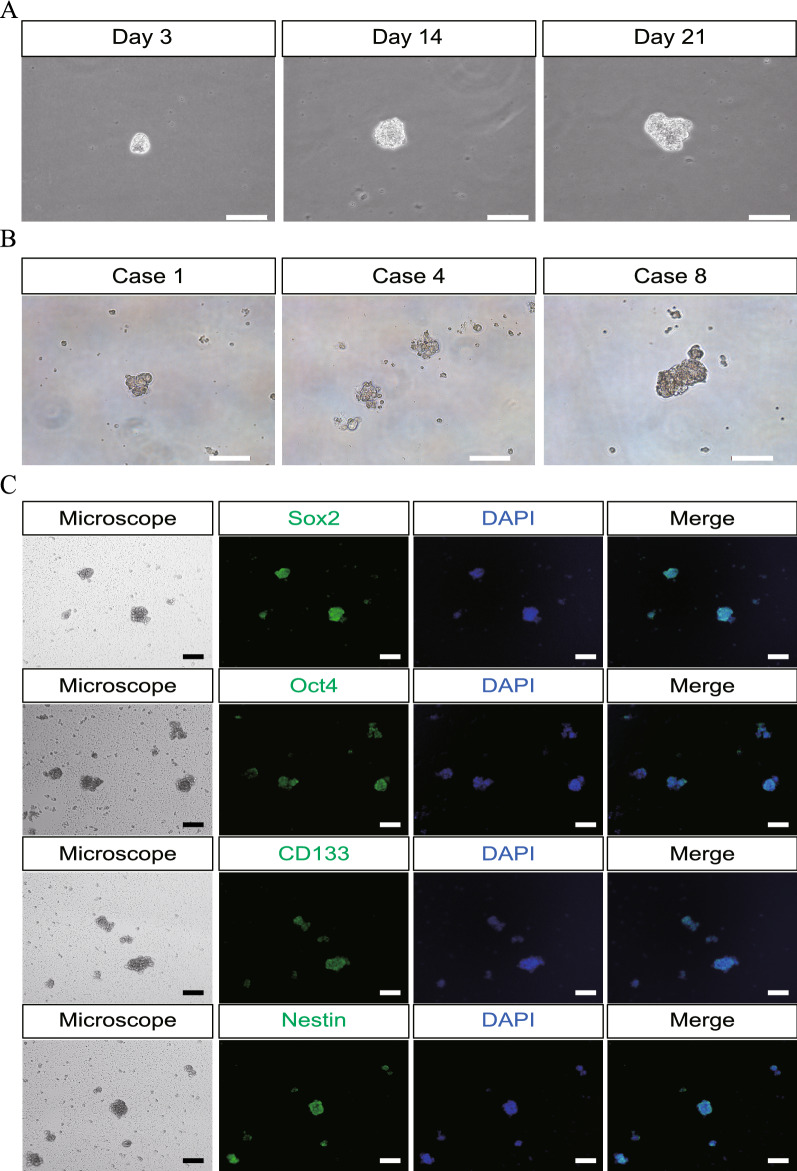
The morphology of human gonadotrophic adenoma stem cells. **A** Representative morphological changes of hPASC on day 3, day 14 and day 21 by microscopic observation from Case 3. **B** Representative morphology of hPASC culture from patient Case 1, Case 4 and Case 8 on day 14. **C** Immunohistochemistry of Sox2, Oct4, CD133 and Nestin in hPASCs cultured on day 14. All the markers were positively stained in the hPASCs. Scale bar is 100um for all panels

Human gonadotrophic pituitary adenoma stem cells were cultured and verified in each sample of 14 patients. The clinical information regarding the included patients is listed in Table 1. On day 0, the tumor cells were completely dissociated into single cells and cultured in serum-free medium supplemented with EGF and bFGF. Small spheres appeared to form after 3 days and became more apparent on day 14. At this point, no other cells such as erythrocytes or fibroblasts were obviously present in the culture by microscopic observation. Figure 1A shows the representative morphological appearance of hPASCs from one patient at different time points. With the addition of a fresh stem culture medium, the spheres continued growing for 21 days. Moreover, the size of the hPASC spheres varied between samples. Figure 1B shows the morphology of hPASC from the tumor samples of three different patients on day 14.

**Table 1:** Summary of clinical information for hPASC culture

	Category	Case (%)
Gender	Male	11 (78.6%)
	Female	3 (21.4%)
Age (years)	30–49	2 (14.3%)
	50–59	7 (50%)
	> 60	5 (35.7%)
Pituitary cell lineage (T-Pit, Pit1, SF-1)	SF-1 positive	14 (100%)
Maximal diameter of the tumor (mm)	10–29	9 (64.3%)
	30–59	4 (28.6%)
	> 60	1 (7.1%)
Knosp grade	1	1 (7.1%)
	2	4 (28.6%)
	3	4 (28.6%)
	4	5 (35.7%)
Ki-67 (%)	1–3	9 (64.3%)
	4–6	4 (28.6%)
	> 7	1 (7.1%)
FSH (mIU/ml) *0.7–11.1	0–11.1	9 (64.3%)
	11.2–19.9	4 (28.6%)
	> 20	1 (7.1%)

Immunostaining was performed to examine the expression of the generic stem cell markers Sox2, Oct4, CD133 and Nestin in the hPASCs cultured in the stem cell medium on day 14. As shown in Fig. 1C, positive staining was observed for all four markers in the cultured hPASCs.

### Differentiation of hPASCs

**Fig. 2:**
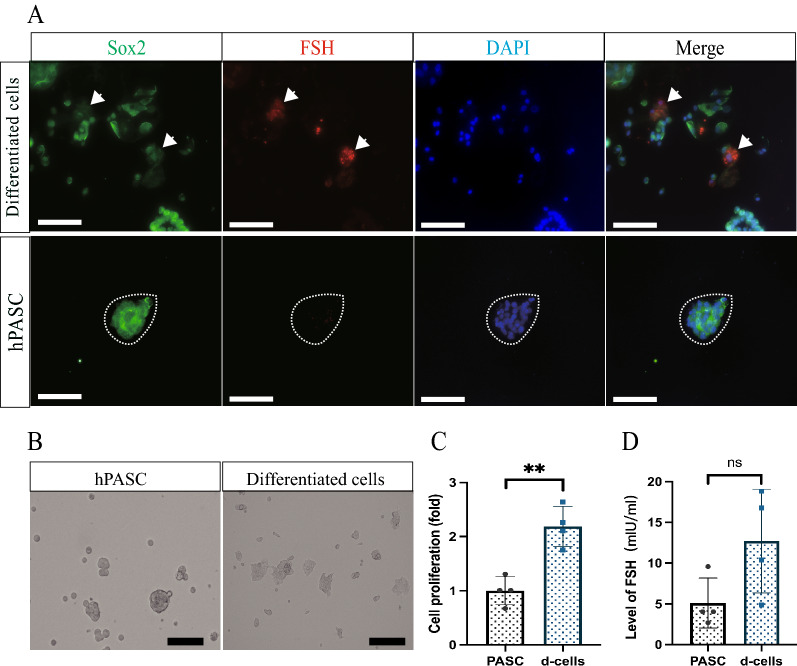
Human gonadotrophic adenoma stem cells possessed the capacity of differentiation. **A** Immunostaining of FSH and Sox2 in hPASCs and the differentiated daughter cells. Compared to the hPASCs, the differentiated cells expressed a much lower level of Sox2 but strong FSH. **B** Representative images of hPASCs and the differentiated cells. **C** Quantification of cell number between hPASCs and the differentiated cells. After 5-day differentiation, the number of cells in the differentiated group was significantly higher than the hPASC group. (**p < 0.005; n = 4 independent experiments). **D** Measurement of FSH level in the hPASC and the differentiated cells by ELISA. The level of FSH in the differentiated cells was not significantly changed compared to the hPASC group (*p* > 0.05; n = 4 independent experiments). d-cells: differentiated cells. Scale bar is 100um for all panels

The differentiation capacity of the cultured hPASCs was examined by supplementing FBS at a final concentration of 15% to the medium. After 5 days of differentiation, the expression level of Sox2 was reduced in differentiated daughter cells compared to the hPASCs. FSHβ, a marker for FSH, was positively stained in the differentiated cells but not the hPASCs (Fig. 2A). After the 5-day differentiation, the number of differentiated daughter cells was significantly increased by 2.19 ± 0.29 (*p* < 0.005) compared to the hPASC (Fig. 2B, C). However, the level of FSH secreted by the differentiated cells into the medium was not significantly changed (Fig. 2D).

### DEG profiles between hPASC and matched tumor samples

**Fig. 3:**
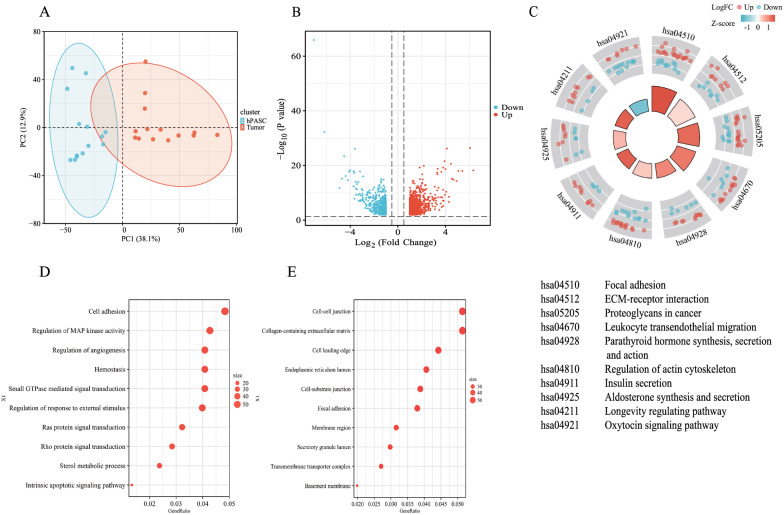
Illustration of differentially expressed genes in hPASC and the matched tumor cells. **A** Principal Components Analysis (PCA) showed a distinctive expression pattern between hPASCs (blue) and the matched tumor cells (red). **B** Volcano plot showed the distribution of the upregulated and downregulated genes presented in hPASCs and the tumor cells. Those upregulated genes (Log_2_FC > 1, *p* < 0.05) were represented as red dots while downregulated ones were in blue (Log_2_FC < -1, *p* < 0.05). **C** KEGG enrichment of differentially expressed genes between hPASC and the matched tumor cells. (**D**-**E**) GO analysis of differentially expressed genes between hPASC and the matched tumor cells

To explore the genetic profiling of gonadotrophic pituitary adenoma stem cells, RNA-sequencing was performed in the hPASCs and the matched tumor samples. Principal component analysis (PCA) showed that both groups manifested a significant distinction regarding the overall expression pattern (Fig. 3A). There were 1352 differentially expressed genes (Log_2_FC > 1 or <  − 1, adjusted *p* < 0.05) between the hPASCs and matched tumor samples. In detail, 843 genes were significantly upregulated (Log_2_FC > 1, adjusted *p* < 0.05) and 509 genes were downregulated (Log_2_FC <  − 1, adjusted *p* < 0.05) in the hPASC group. The volcano plot depicting the expression pattern of these genes is shown in Fig. 3B.

Based on the differentially expressed genes, enrichment analysis was performed to reveal key factors and signaling pathways. The top-rated KEGG pathways were focal adhesion, ECM–receptor interaction and proteoglycans in cancer (Fig. 3C). GO analysis identified key events, including the intrinsic apoptotic signaling pathway, the sterol metabolic process, Rho protein signal transduction, the regulation of angiogenesis, and cell adhesion (Fig. 3D). These molecules were mainly enriched in the basement membrane, transmembrane transporter complex, secretory granule lumen, and endoplasmic reticulum lumen (Fig. 3E).

### Core protein–protein interaction (PPI) network

**Fig. 4:**
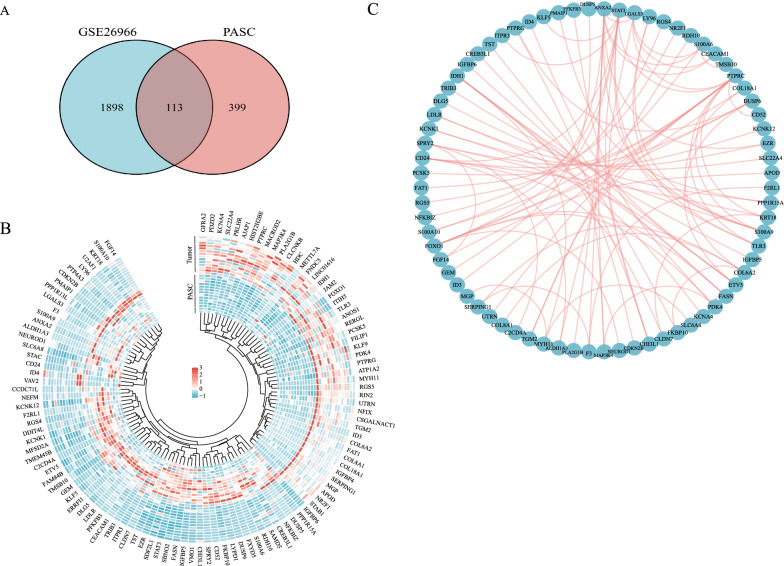
Analysis of shared differentially expressed genes in both GSE26966 dataset and hPASCs. **A** Venn diagram showed 113 differentially expressed genes were exclusively contained in both GSE26966 dataset and RNA-sequencing results of hPASCs. **B** Heatmap of 113 genes expressed in hPASCs and the matched tumor cells. **C** Protein–protein Interaction (PPI) of 113 genes was analyzed by String.org and the network was depicted using cytoscape

GSE26966 dataset contained the expression profiles of 14 gonadotrophic pituitary adenomas compared with 3 normal pituitary tissues. To screen the essential genes altered during the tumorigenesis of hPASCs, 512 of 1352 candidate genes were selected according to specific criteria (Log_2_FC ≥ 1.5 or ≤  − 1.5, *p* < 0.05) through RNA-sequencing and aligned with the genes listed in GSE26966. One hundred and thirteen genes were shared in both datasets (Fig. 4A), and their expression pattern was shown in Fig. 4B between hPASCs and the matched tumors. The PPI network among those genes was predicted using the string database (Fig. 4C).

### Expression of candidate genes in non-invasive and invasive gonadotrophic pituitary adenomas

**Fig. 5:**
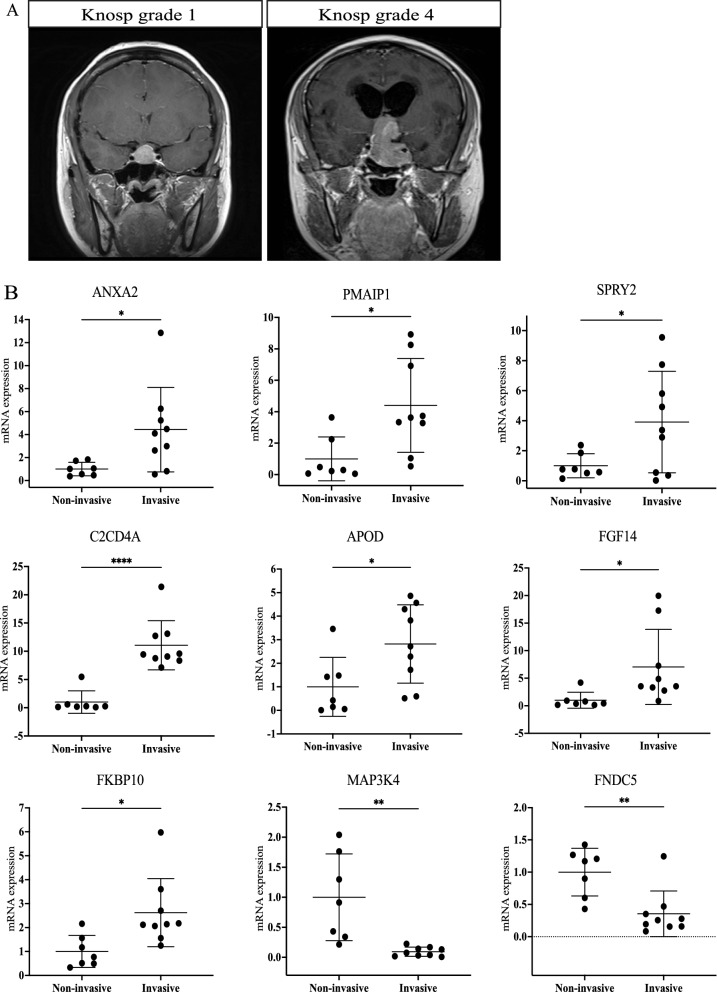
Measurement of gene expression between non-invasive and invasive gonadotrophic pituitary adenoma. **A** MRI images of pituitary adenoma in Knosp grade 1 and Knosp grade 4. **B** The expression of nine candidate genes was quantified and found significantly altered between 7 non-invasive (Knosp grade 1–2) and 9 invasive (Knosp grade 3–4) human gonadotrophic pituitary adenoma clinical samples. *p < 0.05; **p < 0.01; ****p < 0.001

The invasiveness of pituitary adenoma into the surrounding structure indicated the aggressiveness of the tumor. In clinical practice, pituitary adenomas at Knosp grades 3 or 4 are generally considered invasive, while the grade is 0–2 normally for non-invasive tumors (Fig. 5A). Nine genes were significantly differentially expressed between seven non-invasive and nine invasive gonadotrophic pituitary adenoma samples (Fig. 5B). In the invasive gonadotrophic pituitary adenoma (Knosp grade 3–4), seven genes were significantly upregulated (ANXA2, PMAIP1, SPRY2, C2CD4A, APOD, FGF14 and FKBP10) and two genes were downregulated (FNDC5 and MAP3K4).

### Immunohistochemistry staining

**Fig. 6:**
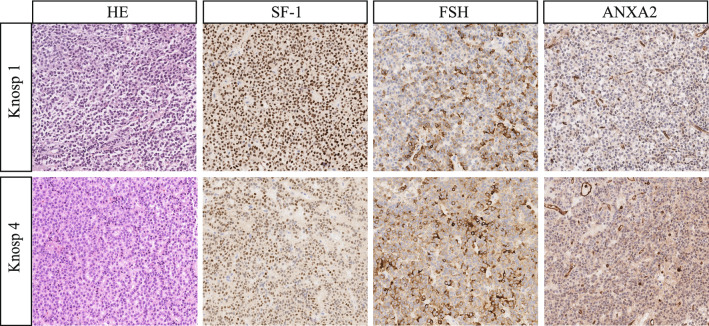
Immunohistochemistry of ANXA2 in non-invasive (Knosp grade 1) and invasive gonadotrophic pituitary adenoma (Knosp grade 4). Magnification scale × 200

The protein level of ANXA2 was examined via immunohistochemical staining of the clinical samples. As shown in Fig. 6, the immunoreactivity of ANXA2 was significantly stronger in the invasive gonadotrophic pituitary adenoma (Knosp grade 4) than in the non-invasive one (Knosp grade 1).

## Discussion

Whole-exome sequencing of silent gonadotrophic pituitary adenoma has detected some putative driver genes such as platelet-derived growth factor-D (PDGFD), N-myc downregulated gene family member 4 (NDRG4), and Zipper sterile-α-motif kinase (ZAK); however, further analysis indicated that these genes do not significantly contribute to tumorigenesis [15]. Other epigenetic factors, such as microRNAs, DNA methylation, and environmental factors were also proposed to explain the molecular mechanism [16–18]. Tumor stem cells have been proposed to explain the tumorigenesis and biological properties of pituitary adenoma for many years. Wurth’s review has summarized convincing evidence of the existence of hPASCs in pituitary adenoma and their potential roles [12]. In this study, hPASCs from gonadotrophic pituitary adenoma were successfully established according to a published protocol with some modifications. To the best of our knowledge, no previous studies have evaluated the genetic profiling of hPASCs in this tumor subtype.

The morphology of the hPASCs forming pituispheres was similar to that reported in the previous literature [10, 11]. The culture time-frame of hPASCs in this study was 21 days, but in our observation, the pituispheres could be expanded continuously to a maximum of 60 days (data not shown). This suggested the self-renewal capacity of these hPASCs. Immunohistochemical staining was commonly used for verification of tumor stem cells in pituitary adenoma; four generic markers, namely Sox2 [11, 19–21], CD133 [22, 23], Nestin [10, 23] and Oct4 [23, 24] were selected and showed immunopositivity in the cultured hPASCs, thus validating the properties of their stemness.

Next, we tested whether the hPASC pituispheres had the capacity to differentiate into tumor cells following the previous protocol [10, 11]. After adding serum to the hPASC culture for 5 days, the total number of cells was significantly increased, indicating that hPASCs could generate some new tumor cells. This is similar to some other types of tumor stem cells like glioblastoma, possibly due to intrinsic alteration of the cell cycle [25]. Moreover, serum supplementation of the gonadotrophic adenoma stem cells not only drove the proliferation but also facilitated the expression of the respective hormone FSH. This was consistent with the previous observation on somatotropinoma stem cells [11]. Although the FSH was expressed in the differentiated cells, ELISA results showed that the level of hormone in the medium from the differentiated cells was not significantly changed. We speculated that the secretive mechanism of the hormone from the cells was not altered during this process, or else the intrinsic molecular properties of the tumor remained unchanged.

Peculis demonstrated that somatic mutations of primary pituitary tumor samples could still be detected in the exomes of respective tumor pituispheres [26], indicating that gene expression, rather than mutations, play a primary role during tumorigenesis. Our RNA-sequencing data from both hPASCs and matched tumor samples have identified the differentially expressed genes during the process. By enrichment analysis, the putative top signaling pathways from KEGG analysis were focal adhesion, ECM–receptor interaction, and proteoglycans in cancer. GO analysis predicted hemostasis, angiogenesis, MAP kinase activity, and cell adhesion involved in the tumorigenesis. Focal adhesion involves many pro-survival signaling molecules, including growth factor receptors, intracellular molecules, and integrins, and plays important roles in the regulation of cell mobility, proliferation, and tumor cell survival [27]. The MAPK pathway has also been widely reported in various studies on pituitary adenoma tumorigenesis [28]. More work is needed to elucidate other potential key pathways during the process of differentiation of hPASCs.

To narrow down the scope of candidate genes, we aligned our RNA-sequencing data with public databases. The rationale was to determine whether the genes that played an essential role in the tumorigenesis co-existed in both databases. The alignment indicated 113 genes, and their interactions can shed a light on the underlying molecular mechanisms of tumorigenesis. Since TSC has been shown to be associated with the aggressiveness of tumors in previous studies [29], we tested the expression of some candidate genes in the invasive and non-invasive gonadotrophic pituitary adenomas. In clinical practice, the Knosp grading system has been widely used in the pituitary adenoma surgery assessment [13]. Some previous studies have already used this system to screen the differences in genetic profiles between invasive and non-invasive pituitary adenomas [30]. Nine genes were verified as being significantly differentially expressed between non-invasive and invasive gonadotrophic pituitary adenomas, and further staining indicates that the gene that produces the ANXA2 protein was one of the key genes. ANXA2 is an important member of the annexin protein family and involved in various cellular functions, including cell division, calcium signaling, and cell growth [31]. Recent studies have identified ANXA2 expression in several cancers where it promotes tumor progression via proliferation, migration, epithelial-mesenchymal transformation (EMT), invasion and stem cell formation [32]. Further analysis of the molecular mechanisms of ANXA2 in the tumorigenesis and invasion of pituitary adenoma is thus needed.

## Conclusion

In this study, we provide insight into the molecular biology of hPASC cultured from human gonadotrophic pituitary adenoma. The presence and differentiation capacity of hPASC were validated in the culture. The genetic profiles of hPASCs and their matched tumor samples were compared. Enrichment analysis identified focal adhesion, ECM–receptor interaction, proteoglycans in cancer, intrinsic apoptotic signaling pathway, sterol metabolic process, Rho protein signal transduction, and regulation of angiogenesis as pivotal pathways involved in the tumorigenesis. There were seven candidate genes significantly increased in the invasive human gonadotrophic pituitary adenoma compared to the non-invasive one. ANXA2 was verified to be one of the key candidate genes in the process of tumor invasiveness. Further studies should be conducted to provide more details on the molecular mechanism of ANXA2 in tumorigenesis and invasiveness. In addition, more therapeutic strategies for treating hPASCs should be developed and assessed, which may reduce tumor invasion and relapse.

## Supplementary Information

**Additional file 1**. **Table S1.** Clinical demographics of gonadotrophic pituitary adenoma patients in hPASC culture and sequencing; **Table S2.** Clinical demographics of invasive and non-invasive gonadotrophic pituitary adenoma patients for Real-time PCR quantification; **Table S3.** PCR primers for verification of 9 candidate genes in invasive and non-invasive gonadotrophic pituitary adenoma.

## Data Availability

The datasets generated and/or analysed during the current study are not publicly available because it is used for further analysis, but are available from the corresponding author on reasonable request.

## Abbreviations

hPASC: Human pituitary adenoma stem cells
FSH: Follicle-stimulating hormone
LH: Leucine hormone
TSC: Tumor stem cells
PBS: Phosphate buffer saline
DMEM: Dulbecco's modified eagle’s medium
bFGF: Basic fibroblast growth factor
EGF: Epidermal growth factor
FBS: Fetal bovine serum
ELISA: Enzyme-linked immunosorbent assay
GO: Gene ontology (GO)
KEGG: Kyoto encyclopedia of genes and genomes (KEGG)
GEO: Gene expression omnibus
PCA: Principal component analysis
PPI: Protein–protein interaction
