# Supplementary material for: Identification and gene expression profiling of human gonadotrophic pituitary adenoma stem cells
Source: Acta Neuropathol Commun. 2023 Feb 7;11:24. doi: 10.1186/s40478-023-01517-w (PMC9906881; doi:10.1186/s40478-023-01517-w)
Supplement: Supplementary file 1 — Additional file 1. Table S1. Clinical demographics of gonadotrophic pituitary adenoma patients in hPASC culture and sequencing; Table S2. Clinical demographics of invasive and non-invasive gonadotrophic pituitary adenoma patients for Real-time PCR quantification; Table S3. PCR primers for verification of 9 candidate genes in invasive and non-invasive gonadotrophic pituitary adenoma. [file 40478_2023_1517_MOESM1_ESM.docx]

Supplemental Table 1. Clinical demographics of gonadotrophic pituitary adenoma patients in hPASC culture and sequencing

| Case | Gender | Age  (years) | Pituitary cell lineage  (T-Pit, Pit1, SF-1) | Maximal diameter of  the tumor (mm) | Knosp  Grade | Ki-67  (%) | FSH  (mIU/ml)  *0.7-11.1 | LH  (mIU/ml) ^#^0.8-7.6 |
| --- | --- | --- | --- | --- | --- | --- | --- | --- |
| 1 | Male | 31 | SF-1 | 39 | 4 | 5 | 9.05 | 0.98 |
| 2 | Male | 57 | SF-1 | 25 | 3 | 8 | 8.28 | 2.61 |
| 3 | Male | 60 | SF-1 | 15 | 2 | 3 | 9.29 | 5.55 |
| 4 | Male | 55 | SF-1 | 69 | 4 | 5 | 17.9 | 2.38 |
| 5 | Male | 66 | SF-1 | 36 | 4 | 3 | 7.41 | 1.7 |
| 6 | Male | 37 | SF-1 | 26 | 3 | 1 | 8.33 | 3.41 |
| 7 | Female | 66 | SF-1 | 48 | 4 | 4 | 19 | 23.9 |
| 8 | Female | 66 | SF-1 | 19 | 2 | 3 | 59.1 | 17.5 |
| 9 | Male | 58 | SF-1 | 13 | 2 | 2 | 19.7 | 5.31 |
| 10 | Male | 58 | SF-1 | 13 | 2 | 2 | 11.5 | 7.61 |
| 11 | Male | 52 | SF-1 | 47 | 4 | 3 | 6.56 | 2.9 |
| 12 | Male | 56 | SF-1 | 15 | 1 | 3 | 3.93 | 2.06 |
| 13 | Female | 57 | SF-1 | 26 | 3 | 5 | 4.49 | 1.07 |
| 14 | Male | 67 | SF-1 | 29 | 3 | 3 | 2.19 | 0.19 |

* Normal range of FSH

# Normal range of LH

Supplemental Table 2. Clinical demographics of invasive and non-invasive gonadotrophic pituitary adenoma patients for Real-time PCR quantification

| Case | Gender | Age  (years) | Maximal diameter of  the tumor (mm) | Knosp Grade | Invasive | Resection |
| --- | --- | --- | --- | --- | --- | --- |
| 1 | F | 44 | 27 | 3 | Y | GTR |
| 2 | M | 57 | 19 | 3 | N | NGTR |
| 3 | M | 51 | 35 | 4 | Y | NGTR |
| 4 | M | 30 | 36 | 3 | Y | NGTR |
| 5 | M | 58 | 47 | 3 | N | NGTR |
| 6 | M | 52 | 19 | 1 | N | GTR |
| 7 | M | 55 | 30 | 2 | N | GTR |
| 8 | F | 69 | 30 | 3 | Y | GTR |
| 9 | F | 65 | 24 | 1 | Y | GTR |
| 10 | M | 63 | 30 | 3 | Y | NGTR |
| 11 | F | 21 | 14 | 1 | N | GTR |
| 12 | M | 55 | 50 | 3 | N | NGTR |
| 13 | M | 42 | 50 | 4 | N | NGTR |
| 14 | M | 57 | 59 | 3 | N | NGTR |
| 15 | F | 44 | 55 | 4 | N | NGTR |
| 16 | F | 56 | 24.93 | 1 | Y | GTR |

GTR：gross total resection; NGTR: non-gross total resection

Supplemental Table 3. PCR primers for verification of 9 candidate genes in invasive and non-invasive gonadotrophic pituitary adenoma

| Sequence Definition | Sense Primer | Anti-sense Primer |
| --- | --- | --- |
| ANXA2 | TATTGCCTTCGCCTACCA | CTTCTCCAGATCAGTCTTGTA |
| PMAIP1 | AGTTGGAGGCTGAGGTTC | GGTTCCTGAGCAGAAGAGT |
| SPRY2 | ATCCATAAGCACGGTCAG | CAGTTGTCCTCATCATCATT |
| C2CD4A | TTGGGTCCTGAGTGCCTTC | TCGTCCATCCCTGCTTCT |
| APOD | GTGAATAAGTATCTCGGAAG | GCAGGTACAGGAATACAC |
| FGF14 | CACTCTTCAACCTCATACCA | AACCAGGCTCTACCAGATT |
| FKBP10 | CACACCTACAATACCTATATCG | AAGATTAGCACGGCAGAG |
| FNDC5 | TGGAGGAGGATACGGAGTA | CAGGACCACGACGATGAT |
| MAP3K4 | CGATGCCTATCTGCTTCT | CTCAATGGACTGCTGGAA |
| beta-actin | ACTTAGTTGCGTTACACCCTT | GTCACCTTCACCGTTCCA |
